# Lack of protective effect of CCR3 blockade during experimental colitis may be related to CCR3 expression by colonic Tregs

**DOI:** 10.1002/ctm2.455

**Published:** 2021-06-27

**Authors:** Maroua Ferhat, Julie Hablot, Mahdia Taieb, Fatouma Salem, Patrick Netter, Laurent Peyrin‐Biroulet, Jean‐Yves Jouzeau, David Moulin

**Affiliations:** ^1^ IMoPA UMR7365 CNRS‐Université de Lorraine Vandœuvre Les Nancy France Université de Lorraine CNRS IMoPA Nancy France; ^2^ NGERE UMR‐U1256 INSERM‐Université de Lorraine Vandœuvre Les Nancy France Université de Lorraine, Inserm NGERE Nancy France; ^3^ Service d'hépato‐gastroentérologie CHRU de Nancy Vandœuvre Les Nancy Nancy France; ^4^ Service de Pharmacologie Clinique et de Toxicologie CHRU‐Nancy Nancy France; ^5^ CHRU de Nancy Contrat d'interface Vandœuvre Les Nancy Nancy France

Dear Editor,

While previous findings indicate that blockade of the chemokine receptor CCR3 can be viewed as an attractive therapeutic alternative to actual treatments in inflammatory bowel diseases (IBD), we demonstrate herein that CCR3 deficiency or pharmacological blockade does not protect and even aggravates clinically, histologically, and biologically DSS‐induced colitis. Interestingly, we show that a subpopulation of colonic regulatory T (Treg) cells express CCR3 providing an explanation for this unexpected deleterious effect of CCR3 inhibition or absence during experimental colitis.

Over the past two decades, several drugs (eg, biologics and tofacitinib) have changed IBD management. However, a significant proportion of patients remain or became refractory to these drugs, indicating an urgent need for new therapeutic targets.

CCR3 is a chemokine receptor, highly expressed by eosinophils and basophils, that have been implicated in autoimmune disorders and allergic diseases. CCR3 binds a wide range of ligands necessary for chemotaxis and tissue infiltration, including RANTES (CCL5), MCP‐3 (CCL7), MCP‐4 (CCL13), MIP‐5 (CCL15), CCL18 (MIP‐4), Eotaxin 1 (CCL11), Eotaxin‐2 (CCL24), Eotaxin‐3 (CCL26), and MEC (CCL28).^1^, ^3^ Among these, eotaxin‐1 is a key regulator of intestinal inflammation as demonstrated experimentally^1^, ^2^ and clinically.^3^ Similarly, eotaxin‐2, is also increased in ulcerative colitis (UC) patients.^4^

All these lines of evidence provide a basis for targeting the eosinophil/eotaxin axis in patients with UC. In this prospect, Bertilimumab, an IgG4 monoclonal antibody that targets eotaxin‐1, with a very high affinity and specificity, was developed and is currently in phase 2 clinical trial for the treatment of moderate to severe UC (NCT01671956).^5^ In addition, anti‐eotaxin‐2 antibodies are currently investigated for their potential use in the treatment of IBD (US Patent # 9,067,989).

Experimental and clinical data indicate that RANTES is also a player of IBD.^6^, ^7^ Moreover, clinical investigations showed an over‐expression of eotaxin‐3^4^, ^8^ and MIP‐2^9^ in the tissue and serum of UC patients.

Therefore, to explore the relevance of CCR3 inhibition in colitis, we first decided to block CCR3 using GW766994, a CCR3 selective antagonist, during DSS‐induced colitis in mice (Figure 1A). Surprisingly, GW766994 treatment was associated with a higher incidence rate (Figure 1B) and a more severe disease as assessed by DAI (disease activity index), body‐weight loss and survival rate (Figure 1C‐E) compared to DSS control mice.

**FIGURE 1:**
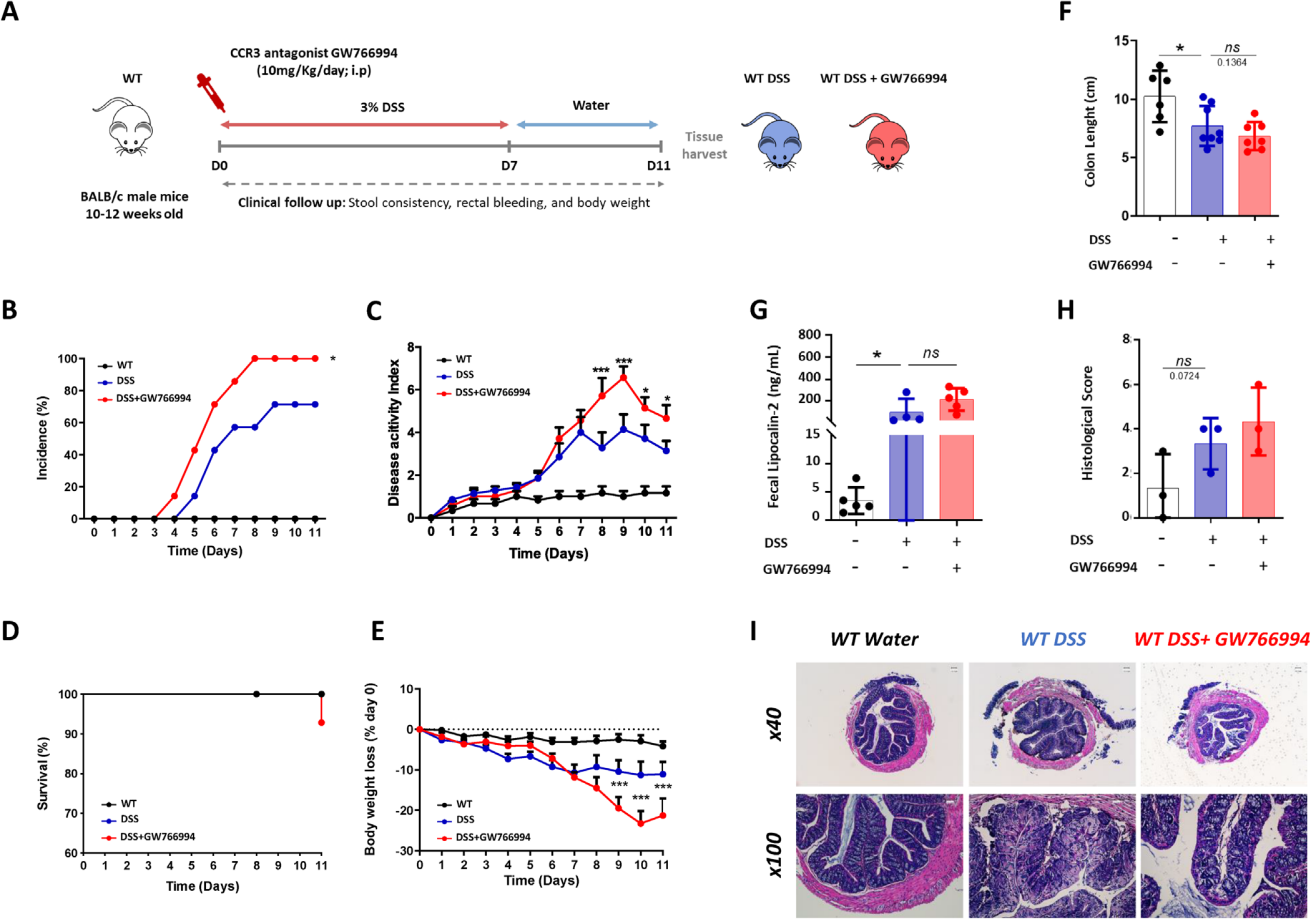
Lack of protective effect of CCR3 pharmacological inhibition in dextran sodium sulfate (DSS)‐induced colitis. A, Schematic representation of study design: 10‐12‐week‐old BALB/c were challenged with 7 days of 3% DSS in drinking water, followed by a 4 day of washout phase. During all the procedures, mice were receiving (i.p) a daily dose (10 mg/kg) of CCR3 antagonist GW766994 or saline alone. B, Incidence of colitis C‐E, Disease activity index (DIA) (C), survival rates (D), and bodyweight variation relative to baseline (% of day 0) of mice challenged with DSS. (E) have been recorded in control and colitic mice (n = 8 per group). Mice were killed on day 11 and colon length (F) was measured and feces were collected to asses Lipocalin‐2 level (G). H, Histologic score of one representative experiment based on hematoxylin and eoxin (HE) staining. I, HE staining of distal colon harvested on day 11 of DSS treatment. Each panel is representative of tissue from at least 3 mice (x40 magnification and 100× magnification). Data were pooled from 2–3 independent experiments. (n = 5–9/group). Data are expressed as mean ± SEM, Two‐way ANOVA or ANOVA with Tukey *posthoc* test for multiple comparisons were used for statistical differences between groups. n.s: non‐significant; ^*^ *P* < .05; ^**^ *P* < .01; and ^***^ *P* < .001

Moreover, colon length reduction or fecal lipocalin levels, two markers of gut inflammation remained unaffected by CCR3 antagonist treatment (Figure 1F and G). Colons of GW766994‐treated mice showed similar histological damages (crypts, architecture loss, and leukocytes infiltration) to non‐treated DSS mice (Figure 1H, 1I).

To challenge the results obtained with a pharmacological approach, we used a genetic CCR3 deficiency model. DSS was therefore induced in WT and CCR3 KO BALB/c mice (Figure 2A). Disease incidence (Figure 2B) and severity (Figure 2C) was significantly higher in CCR3‐deficient mice than in WT mice following DSS administration. Such disease worsening was also reflected by a higher body‐weight loss and a trend to a lower survival rate (Figure 2D,E) in CCR3 KO mice compared to WT mice. Moreover, shortening of the colon and increase of fecal lipocalin‐2 level confirmed these findings (Figure 2F,G). Finally, histological examination of CCR3 KO mice showed slightly higher tissue damages in response to DSS, as observed for GW766994‐treated mice (Figure 2H,I). Taken together, these results show that CCR3 KO mice are prone to develop a more severe DSS colitis, suggesting a protective role for CCR3 during colitis.

**FIGURE 2:**
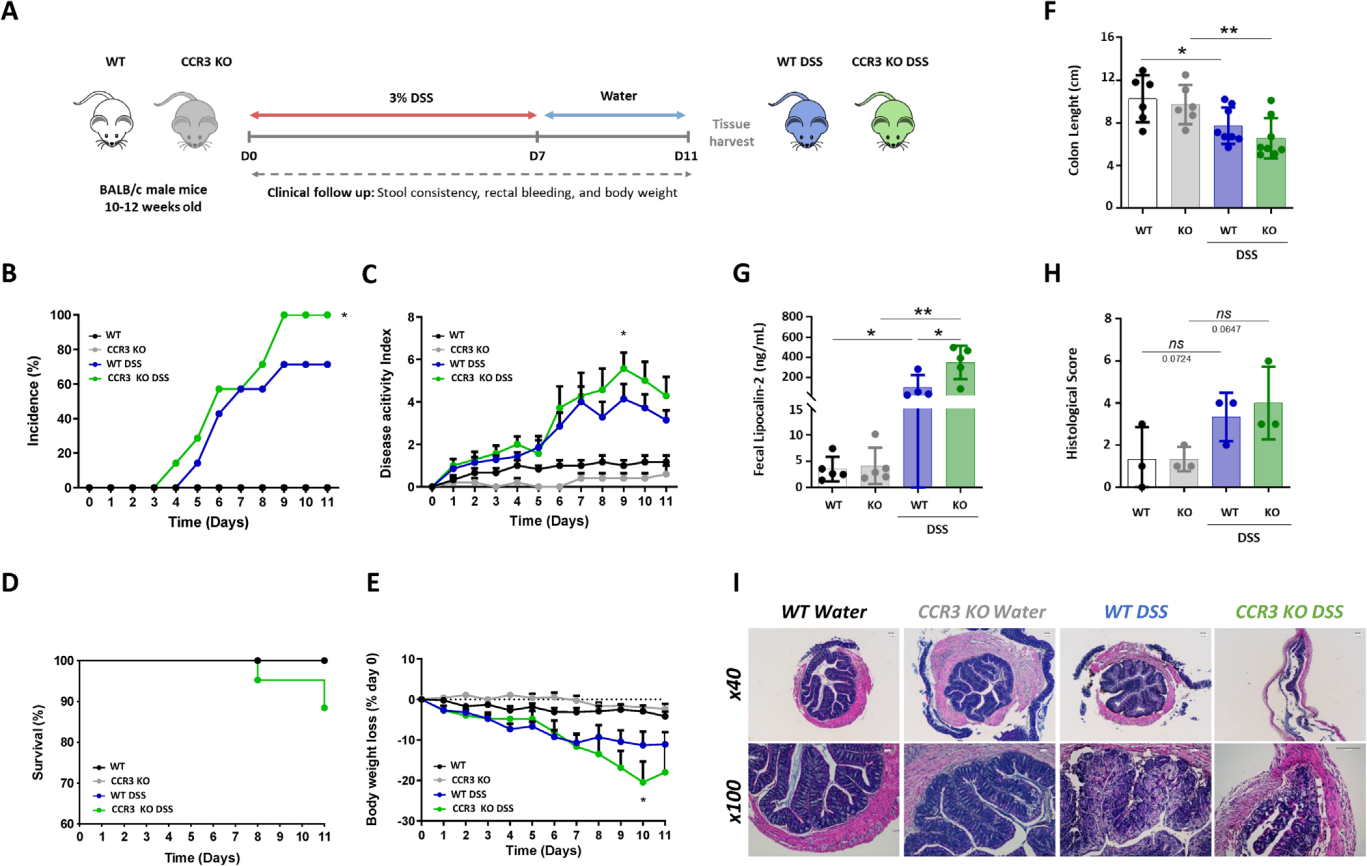
CCR3 genetic deficiency aggravates dextran sodium sulfate (DSS)‐induced colitis. A, Schematic representation of study design: 10‐12‐week‐old BALB/c WT and CCR3 ‐deficient male mice (CCR3 KO) were challenged with 7 days of 3% DSS in drinking water, followed by a 4 day of washout phase. B, Incidence of colitis (C‐E) Disease activity index (DIA) (C), survival rates (D) body‐weight variation relative to baseline (% of day 0) of mice challenged with DSS (E) have been recorded in control and colitic mice (n = 8 per group). Mice were killed on day 11,colon length (F) was measured, and feces were collected to asses Lipocalin‐2 level (G). H, Histologic score of one representative experiment based on hemotoxylin and eoxin (HE) staining. I, HE staining of distal colon harvested on day 11 of DSS treatment. Each panel is representative of tissue from at least three mice (x40 magnification and 100× magnification). Data were pooled from two to three independent experiments. (n = 5–9/group). Data are expressed as mean ± SEM, Two‐way ANOVA, or ANOVA with Tukey *posthoc* test for multiple comparisons used for statistical differences between groups. n.s: non‐significant; *P < .05; **P < .01

T‐helper lymphocytes expressing IL‐17 (Th17) and regulatory T cells (Treg) are critical regulators of intestinal barrier function and key players in colitis. To investigate the contribution of CCR3 to DSS colitis susceptibility, we performed flow cytometric analysis of Th17/Treg population in colon and mesenteric lymph nodes (mLNs). No difference in frequency or absolute number of Th17 relative to CCR3 deficiency or blockade was found (Figure S1), suggesting that DSS polarizes Th17 response independently of CCR3.

Interestingly, while in basal conditions, both WT and CCR3‐deficient mice (left panels) had the same frequency (Figure 3B) and absolute cell number (Figure 3C) of Tregs, abundance of Treg cells was higher in colonic *lamina propria* from WT mice than in CCR3 KO mice (Figure 3C, left panel) after DSS‐challenge. Similar results, were obtained for GW766994‐treated animals in comparison to DSS‐non treated control mice (Figure 3B,C, right panels). In mLNs, frequency and absolute number of CD3^+^ CD4^+^ FoxP 3^+^ Treg cells were significantly decreased in CCR3 KO mice and GW766994‐treated mice (Figure 3D,E, right panels) compared to WT control mice. Consequently, a decrease of Treg over Th17 cells ratios was observed in CCR3 KO mice and GW766994‐treated mice in comparison to control mice, particularly in mLNs (Figure S2B). Our data show that CCR3 deficiency or blockade suppresses Treg response in the large intestine and mLNs of mice, suggesting that CCR3 limits gastrointestinal inflammation *via* the control of Treg cell abundance in these tissues.

**FIGURE 3:**
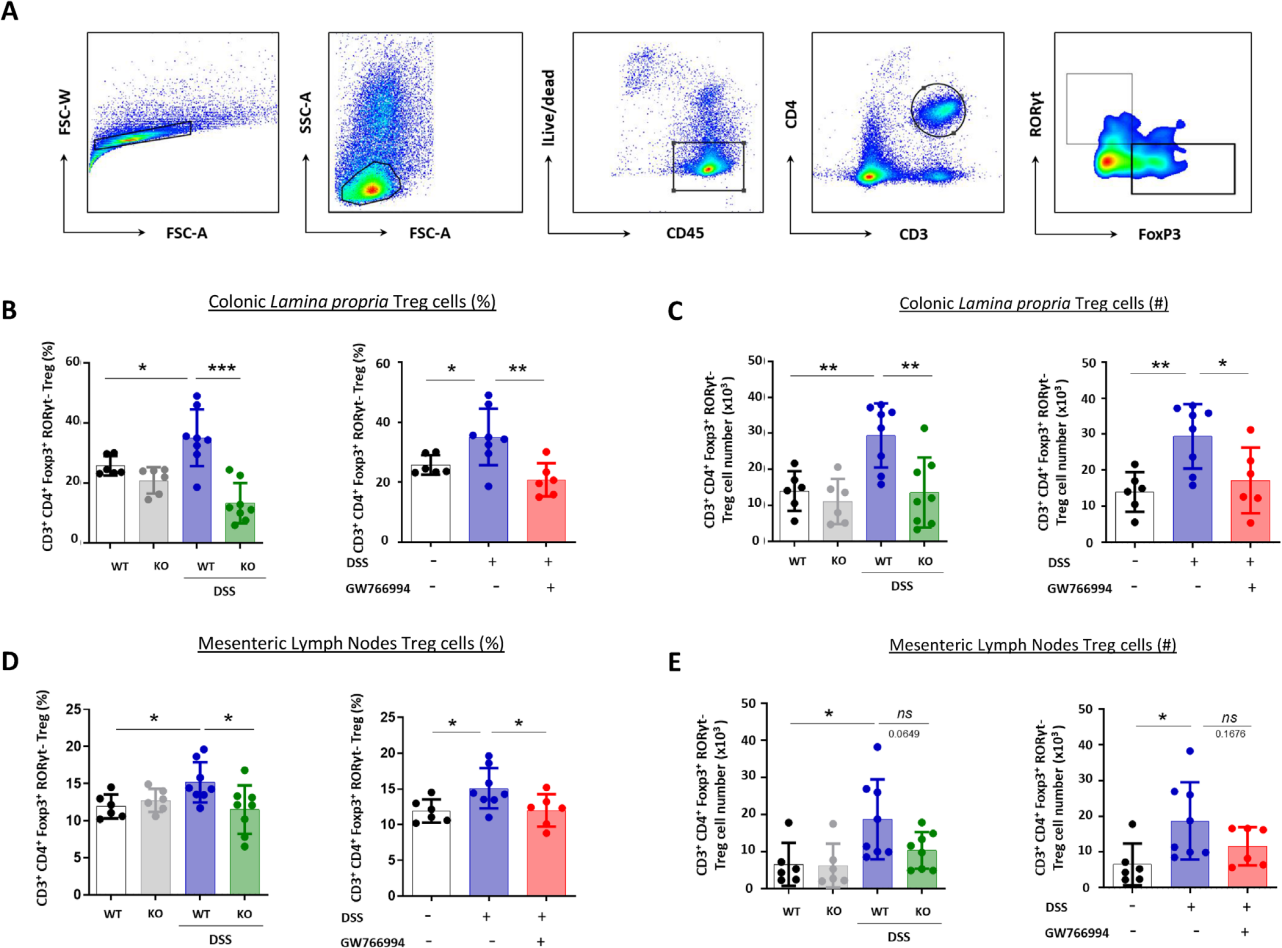
Regulatory T cell (Treg) population is impaired by CCR3 inhibition in DSS‐induced colitis. A, Flow cytometry dot plots showing Treg gating strategy. B, Frequency of CD3^+^ CD4^+^ FoxP3^+^ RORgt^‐^Treg cells isolated from colonic *lamina* *propria* of mice challenged or not with DSS. C, Treg cell count in colonic *lamina* *propria* of mice challenged or not with DSS. D, Frequency of CD3^+^ CD4^+^ FoxP3^+^ Treg cells isolated from mesenteric lymph nodes (mLNs) of mice challenged or not with DSS. E, Treg cell count in mLN of mice challenged or not with DSS. (n = 6‐9/group). Data are expressed as mean ± SEM, ANOVA with Tukey *posthoc* test for multiple comparisons was used for statistical differences between groups. n.s: non‐significant; *P < .05 and **P < .01

As indicated in Figure 4A, Immgen database indicated that CCR3 is expressed by Treg FoxP 3^+^ specifically amongst T cells. We thus analyzed CCR3 expression on Treg cells by flow cytometry. A shown in Figure 4, a fraction of colonic resident and mLNs Treg cells (Figure 4B and 4C) express CCR3 at their cell surface under physiological conditions. Such CCR3 expression is consistent with the diminished level of Treg cells observed in colonic *lamina propria* and mLNs of both CCR3‐deficient and GW‐766994‐treated DSS mice. Since some *lamina propria* Treg cells express RORγt and differ in their function from “classical” Tregs, we investigated the consequence of CCR3 blockade/deletion on these colonic RORγt^+^ Treg. Our results indicate that most of the colonic CCR3 positive Tregs were classical RORγt^‐^ Treg, and that CCR3 blockade or deletion did not affect the RORγt^+^ Treg population (Figure 4D and 4E).

**FIGURE 4:**
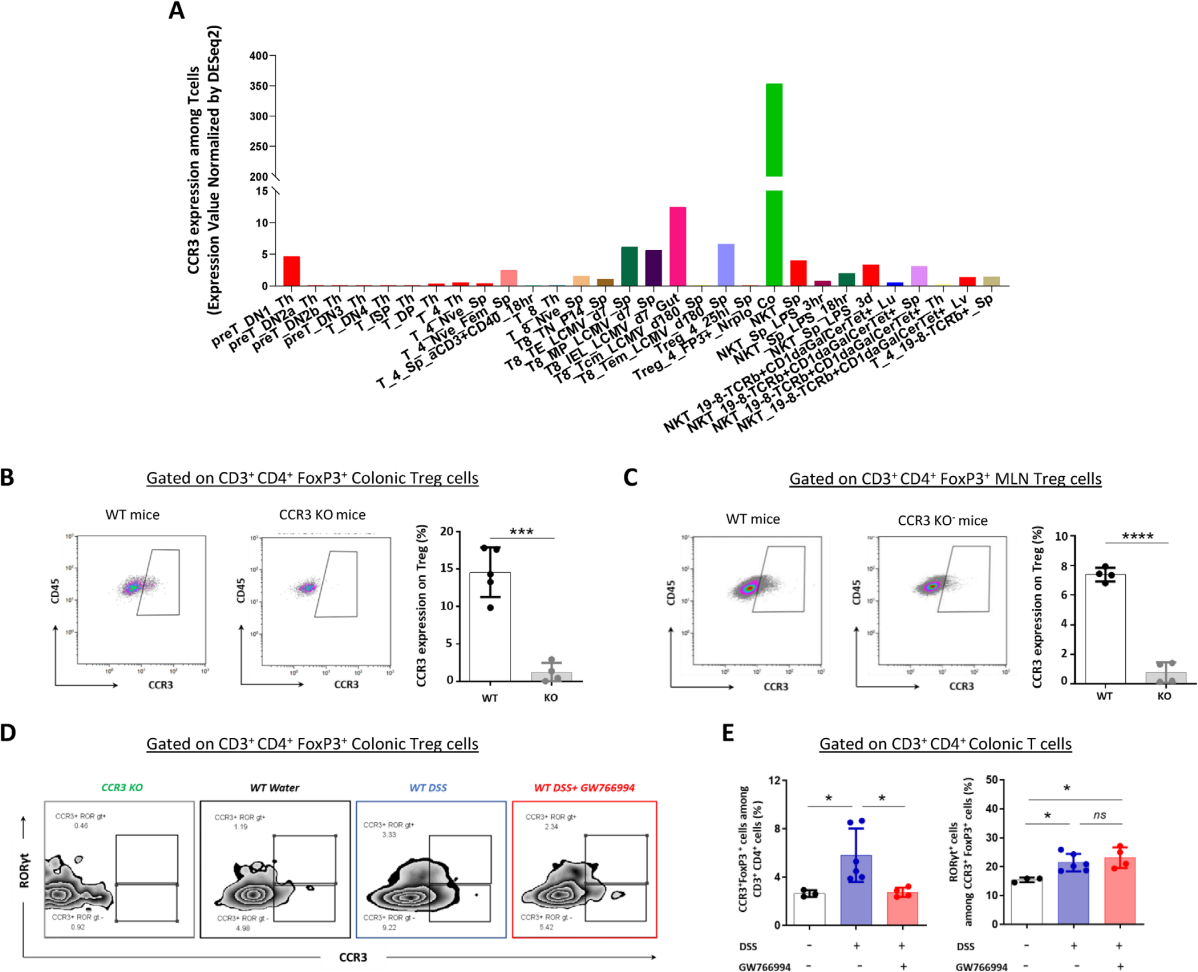
Colonic regulatory T cells (Treg) express CCR3 receptor. A, Immune cell expression of CCR3 according to Immgen data base (www.immgen.org). B, Frequency of CD3^+^ CD4^+^ FoxP3^+^ RORgt^‐^Treg cells isolated from colonic *lamina* *propria* of healthy WT and CCR3 KO mice. Representative dot plots of CCR3 expression on colonic *lamina* *propria* Tregs isolated from healthy WT and CCR3 KO mice. CCR3 expression on colonic *lamina* *propria* Tregs isolated from healthy WT and CCR3 KO mice. C, Frequency of CD3^+^ CD4^+^ FoxP3^+^ Treg cells isolated from mesenteric lymph nodes (mLNs) of healthy WT and CCR3 KO mice. Representative dot plots of CCR3 expression on mLN Tregs isolated from healthy WT and CCR3 KO mice). D, Dot plot of RORγt and CCR3 staining in Foxp3 colonic Tregs. E, Frequency of RORγt^+^ Treg in lamina propria of mice challenged or not with DSS and treated with GW766994. Data are expressed as mean ± SEM, ANOVA with Tukey *posthoc* test for multiple comparisons was used for statistical differences between groups. n.s: non‐significant; ***P < .001; and ****P < .001

Previous reports indicate that CCR3 receptor is also expressed by splenic and adipose tissue Treg cells.^10^ Taking altogether, our results support an immunomodulatory role of CCR3 in shaping the intestinal immune response through Treg cell population in intestinal tissues.

Although all strains were housed in the same animal facility and all precautions were taken to normalize the microbiota of the different strains, we cannot exclude that variation in microbiota composition between strains could have contributed to the phenotypes observed.

In conclusion, our findings do not support the development of CCR3 blockers as potential therapeutic targets for human IBD and shed a new light on Treg cells contribution to the pathogenesis of IBD.

## AUTHOR CONTRIBUTION

P.N., L.P.‐B., J.‐Y.J., and D.M. designed the study and supervised the project; J.H. and M.F. designed parts of the study and performed most of the experiments with the help of M.T. and F.S.; M.F. wrote the manuscript; D.M., J.‐Y. J., L.P.‐B., edited the manuscript.

## DATA AVAILABILITY STATEMENTS

The data underlying this article are available in the article and in its online supplementary material.

## Supporting information

Supporting Information

Supplemental Figure 1. CRR3 inhibition does not exacerbate Th17 cell population after DSS challenge. (A) Flow cytometry dot plots showing Th17 gating strategy. (B) Frequency of CD3^+^ CD4^+^ RORγt^+^ Th17 cells isolated from colonic *lamina* propria of mice challenged or not with DSS. (C) Th17 cell count in colonic *lamina* propria of mice challenged or not with DSS. (D) Frequency of CD3^+^ CD4^+^ RORγt^+^ Th17 cells isolated from mesenteric lymph nodes (mLN) of mice challenged or not with DSS. (E) Th17 cell count in mLN of mice challenged or not with DSS. (n = 3/group). Data are expressed as mean ± SEM, ANOVA with Tukey posthoc test for multiple comparisonswas used for statistical differences between groups. n.s: non‐significant; * p < 0.05; ** p < 0.01 and *** p < 0.001.
Supplemental Figure 2. CCR3 inhibition impairs Treg/Th17 balance in DSS‐induced colitis. (A) Treg/Th17 ratio in colon lamina *propria* of mice challenged or not with DSS. (B) Treg/Th17 ratio in mesenteric lymph nodes (mLN) of mice challenged or not with DSS. (n = 3/group). Data are expressed as mean ± SEM, ANOVA with Tukey posthoc test for multiple comparisonswas used for statistical differences between groups. n.s: non‐significant; * p < 0.05; ** p < 0.01 and *** p < 0.001.
