# Supplementary material for: Lack of protective effect of CCR3 blockade during experimental colitis may be related to CCR3 expression by colonic Tregs
Source: Clin Transl Med. 2021 Jun 27;11(6):e455. doi: 10.1002/ctm2.455 (PMC8236119; doi:10.1002/ctm2.455)
Supplement: Supplementary file 1 — Supporting Information [file CTM2-11-e455-s002.docx]

**Suplementary file**

**Lack of protective effect of CCR3 blockade during experimental colitis may be related to CCR3 expression by colonic Tregs**

Maroua FERHAT^1^*, Julie HABLOT^1^*, Mahdia TAIEB^1^, Fatouma SALEM^1^, Patrick NETTER^1^, Laurent PEYRIN-BIROULET^2,3^, Jean-Yves JOUZEAU^1,4#^ and David MOULIN^1,5#^

1 IMoPA, UMR7365 CNRS-Université de Lorraine, Vandœuvre Les Nancy, France

Université de Lorraine, CNRS, IMoPA, F-54000 Nancy, France

2 NGERE, UMR-U1256 INSERM-Université de Lorraine, Vandœuvre Les Nancy, France

Université de Lorraine, Inserm, NGERE, F-54000 Nancy, France

3 Service d’hépato-gastroentérologie, CHRU de Nancy, Vandœuvre Les Nancy, France

4 Service de Pharmacologie Clinique et de Toxicologie, CHRU-Nancy, Nancy, France

5 CHRU de Nancy, Contrat d’interface, Vandœuvre Les Nancy, France

* These authors contributed equally to this work

# These authors contributed equally to this work

Running title: CCR3 blockade aggravates DSS colitis

Adress for correspondence:

David MOULIN

IMoPA, UMR7365 CNRS-Université de Lorraine, Vandœuvre Les Nancy, France

david.moulin@univ-lorraine.fr to whom requests **for reprints should be addressed**

**+33 3 72 74 65 61**

**Material and methods**

**2.1 Mice.** Age-matched BALB/c wild-type (WT) and CCR3-deficient (CCR3 KO; from the Jackson Laboratory; JAX stock #005440) ^15^ male mice were housed (up to five individuals) in solid-bottomed plastic cages with free access to water and food *ad libitum*. Experiments for this study were approved by the French Committee of Ethics on Animal Experiment (agreement APAFIS10048-2017050311316358). Study designs are summarized in Figure 1A and Figure 2A.

**2.2 Induction and evaluation of colitis.** Acute colitis was induced with 3% (w/v) DSS (molecular weight 36000–50000, MP Biomedicals, US) dissolved in drinking water for 7 days, followed by regular water from day 7 until end of the experiment (day 11). Body weight, stool consistency and rectal bleeding were determined every day. An established composite scoring system including intestinal bleeding was used as an index of disease activity ^16^. The weight change during the experiment was calculated as the percent change in weight compared with the baseline measurement.

**2.3 Treatment regimen.** From day 0 to day 11, one group of DSS colitis mice was administered daily 10mg/kg/d the CCR3 antagonist GW766994 by intra-peritoneal injection. Control DSS mice received the same volume of vehicle only (NaCl 0.9%). GW766994 is an amino-methyl-morpholine derivative able to inhibit CCR3 in the nanomolar range with a good selectivity over alternate targets and no significant inhibition of P-450 cytochromes ^17^. A previous PK/PD studies in rodent model showed that an intraperitoneal dose of 8mg/kg blocked more than 99% of CCR3 receptors after 8 hours and 11% after 24 hours ^18^.

**2.4 Histological analysis.** Following euthanasia on day 11, distal colons were dissected, washed in saline PBS1X and fixed in 10% formalin. The tissues were then embedded in paraffin and sectioned to 5 μm thickness. Hematoxylin and eosin (HE) staining was performed automatically following standard protocols, while images were acquired using a Leica microscope (DMD108). Based on a described scoring system, each tissue was graded for inflammatory infiltration (mucosa, submucosa and muscularis/serosa) and for epithelial and mucosal damages ^19^.

**2.5 Fecal lipocalin-2 assay.** Fresh stools were collected and stored at -80°C until used. Fecal Lipocalin-2 was assessed by using ELISA Kit (MLCN20, R&D Systems, Bio-Techne SAS) according to the manufacturer’s protocol. Samples were diluted 100 times (1:100) for healthy mice and 10 000 times (1:10 000) for DSS-treated mice (assay range 78.1-5,000 pg/mL; lower limit of detection 8.8 pg/mL).

**2.6 Leukocytes isolation.** Mouse colons, mesenteric lymph nodes (mLNs) and spleens, were harvested at sacrifice. Colons were cut longitudinally and washed with cold PBS1X to fully remove colonic content. Mucus and epithelium were removed by two 20 min washes at 37 °C in calcium-free PBS containing 5 mM EDTA. The remaining tissues were excised into fine pieces and washed in PBS, and then digested enzymatically in complete DMEM medium containing DNase (D4527, Sigma-Aldrich) and Liberase T (5401020001, Sigma-Aldrich) at 37 °C for 30 min. Cells and remaining tissues were minced through a 70µm strainer and then a 40µm strainer, washed with complete DMEM medium and resuspended in 40% Percoll (GE Healthcare, US) in complete DMEM medium. Cell suspension were laid onto 80% Percoll and centrifuged at 1470 g for 15 min at RT without braking. *Lamina propria* mononuclear cells were collected at the interface and used for flow cytometry. mLNs and spleens were minced through a 70µm strainer, washed with complete DMEM medium and centrifuged at 500g for 5 min. The pellet was resuspended in staining buffer and put on ice until use.

**2.7 Flow cytometry.** Cells freshly isolated from colons, mesenteric lymph nodes (mLNs) and spleens of WT and CCR3 KO mice were incubated with an FcγR-blocking mAb, and a viability dye and then stained with mAbs against surface markers: CD45 (30-F11), CD3 (17A2) , CD4 (RM4-5), CD19 (6D5) and CCR3 (REA122) for 30 min at 4 °C. Cells were washed, fixed and permeabilized (00-5523-00, eBioscience) for 30 min at room temperature (RT) and then stained for intranuclear factors using mAbs against mouse FoxP3 (150D) and RORγt (AFKJS-9) for 1h at 37 °C. Antibodies for flow cytometry analysis were purchased from BD Biosciences (San Diego, CA, USA) or BioLegend (San Diego, CA, USA), except for CCR3 obtained from Milteny Biotec (Germany). Data were recorded using Gallios flow cytometer (Beckman Coulter) and analyzed using Kaluza software.
