# Supplementary material for: Lack of protective effect of CCR3 blockade during experimental colitis may be related to CCR3 expression by colonic Tregs
Source: Clin Transl Med. 2021 Jun 27;11(6):e455. doi: 10.1002/ctm2.455 (PMC8236119; doi:10.1002/ctm2.455)

Fig 1

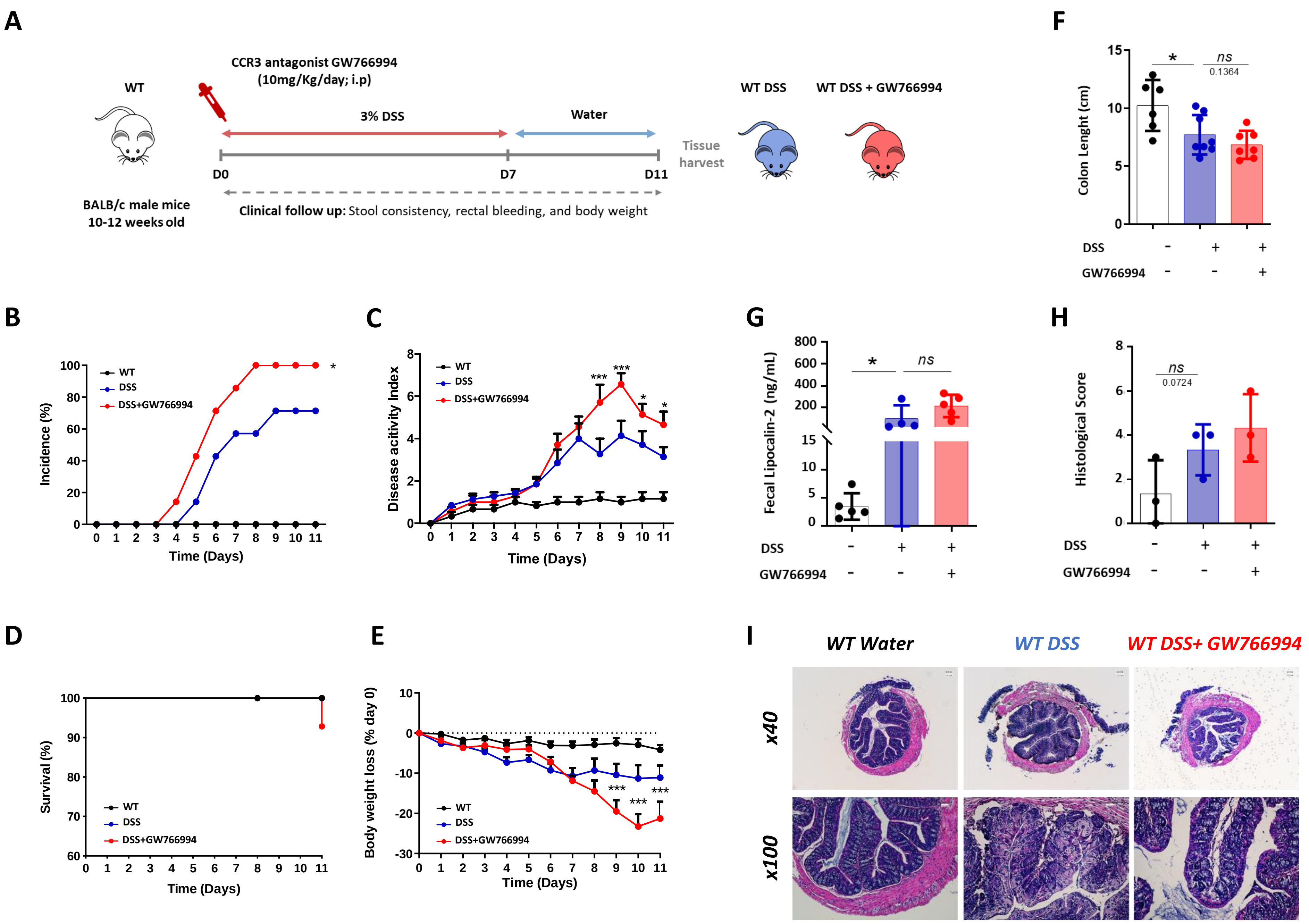
A
WT
BALB/c male mice
10-12 weeks old
CCR3 antagonist GW766994
(10mg/Kg/day; i.p)
3% DSS
Water
D0
D7
D11
Clinical follow up: Stool consistency, rectal bleeding, and body weight
Tissue harvest
WT DSS
WT DSS + GW766994
B
Incidence (%)
Time (Days)
WT
DSS
DSS+GW766994
C
Disease acitivity Index
Time (Days)
D
Survival (%)
Time (Days)
E
Body weight loss (% day 0)
Time (Days)
F
Colon Lenght (cm)
ns
0.1364
DSS
GW766994
G
Fecal Lipocalin-2 (ng/mL)
ns
H
Histological Score
ns
0.0724
I
WT Water
WT DSS
WT DSS+ GW766994
x40
x100

Fig 2

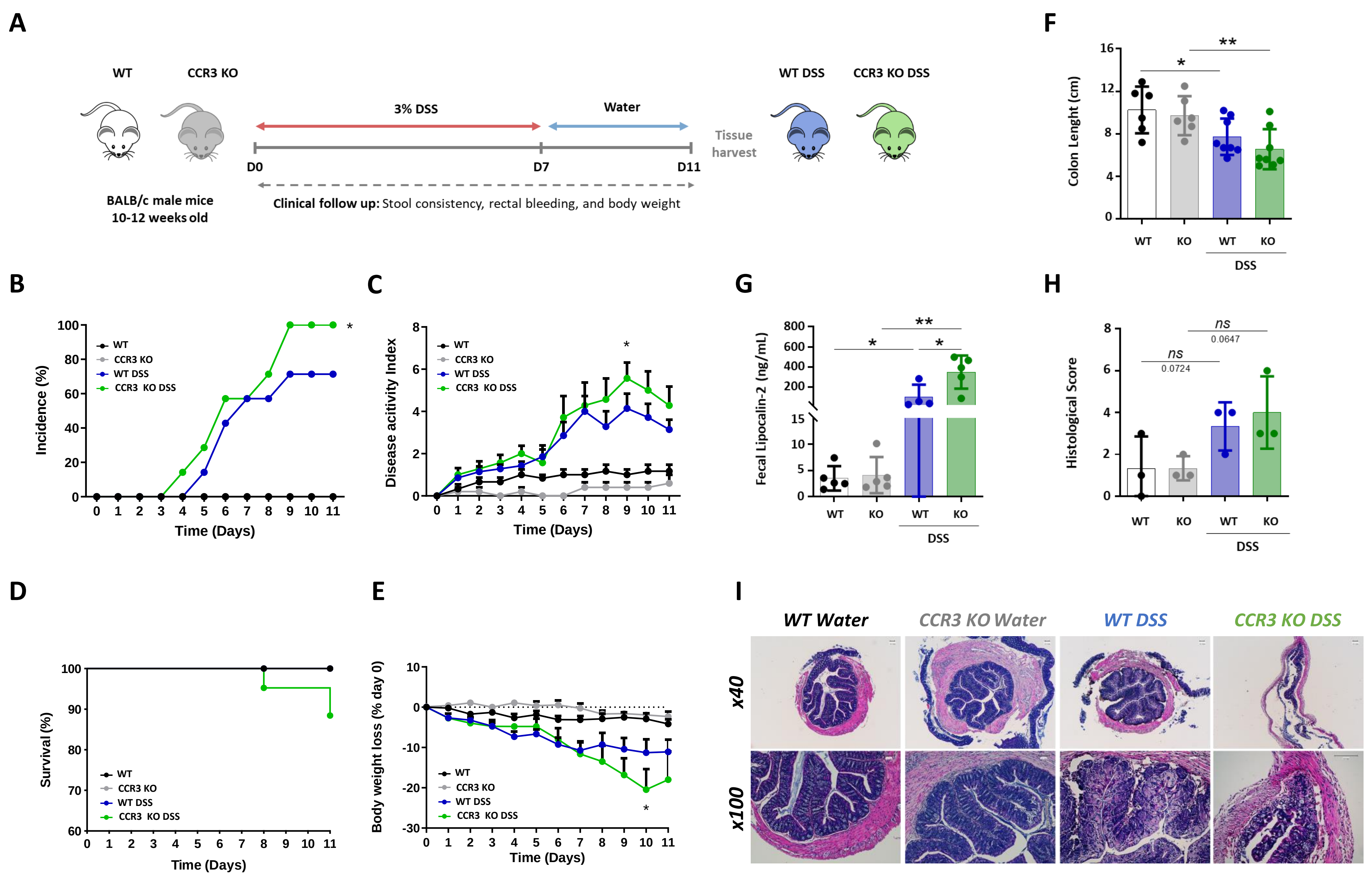

A
WT
CCR3 KO
3% DSS
Water
D0
D7
D11
Tissue harvest
WT DSS
CCR3 KO DSS
BALB/c male mice
10-12 weeks old
Clinical follow up: Stool consistency, rectal bleeding, and body weight
B
Incidence (%)
Time (Days)
WT
CCR3 KO
WT DSS
CCR3 KO DSS
C
Disease acitivity Index
Time (Days)
D
Survival (%)
Time (Days)
E
Body weight loss (% day 0)
Time (Days)
F
Colon Lenght (cm)
WT
KO
DSS
G
Fecal Lipocalin-2 (ng/mL)
H
Histological Score
ns
0.0724
0.0647
I
WT Water
CCR3 KO Water
WT DSS
CCR3 KO DSS
x40
x100

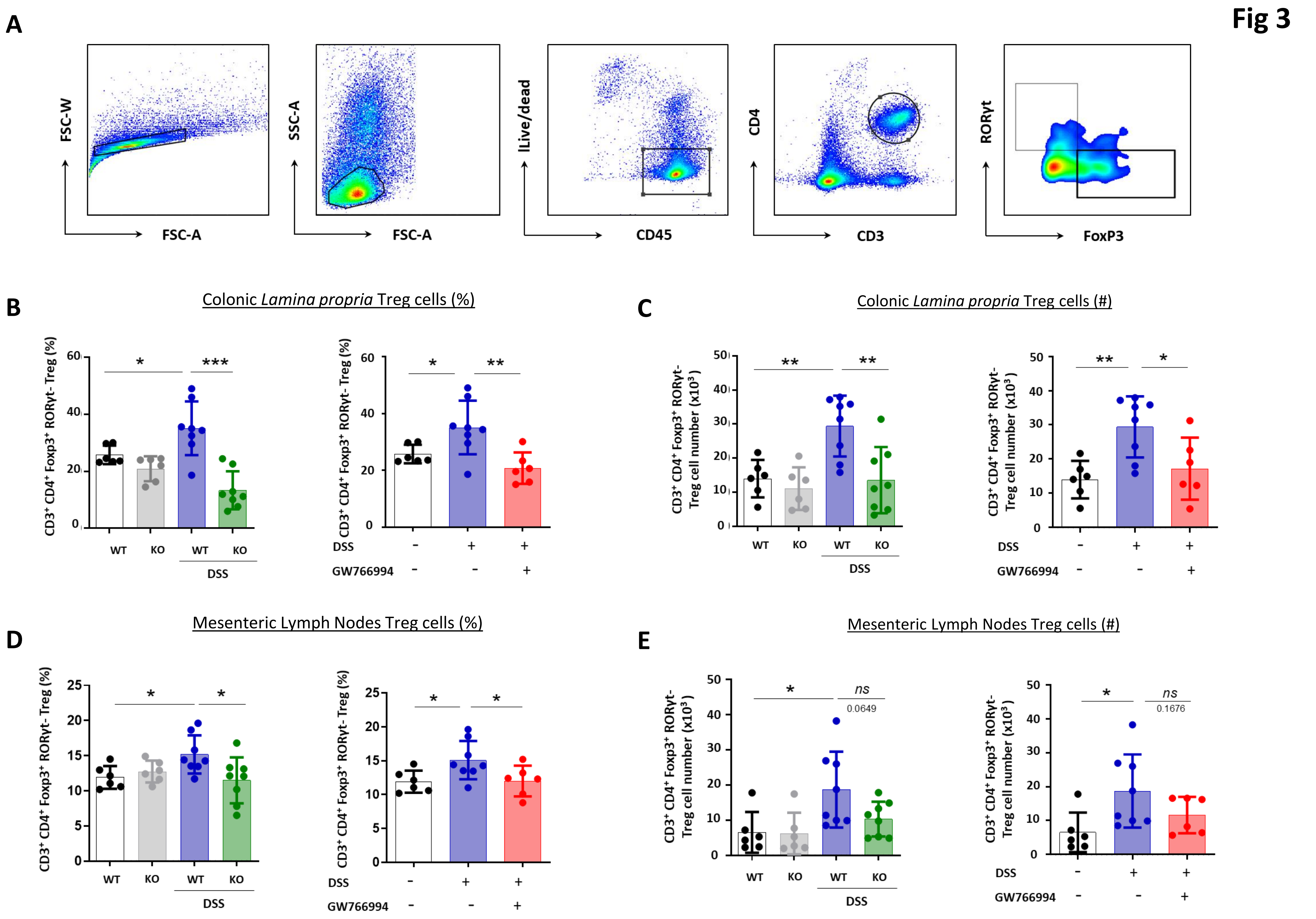

Fig 3
A
FSC-W
FSC-A
SSC-A
FSC-A
lLive/dead
CD45
CD4
CD3
RORγt
FoxP3
B
Colonic Lamina propria Treg cells (%)
CD3+ CD4+ Foxp3+ RORγt- Treg (%)
WT
KO
WT
KO
DSS
GW766994
C
Colonic Lamina propria Treg cells (#)
CD3+ CD4+ Foxp3+ RORγt- Treg cell number (x10³)
D
Mesenteric Lymph Nodes Treg cells (%)
E
Mesenteric Lymph Nodes Treg cells (#)
ns
0.0649
0.1676

Fig 4

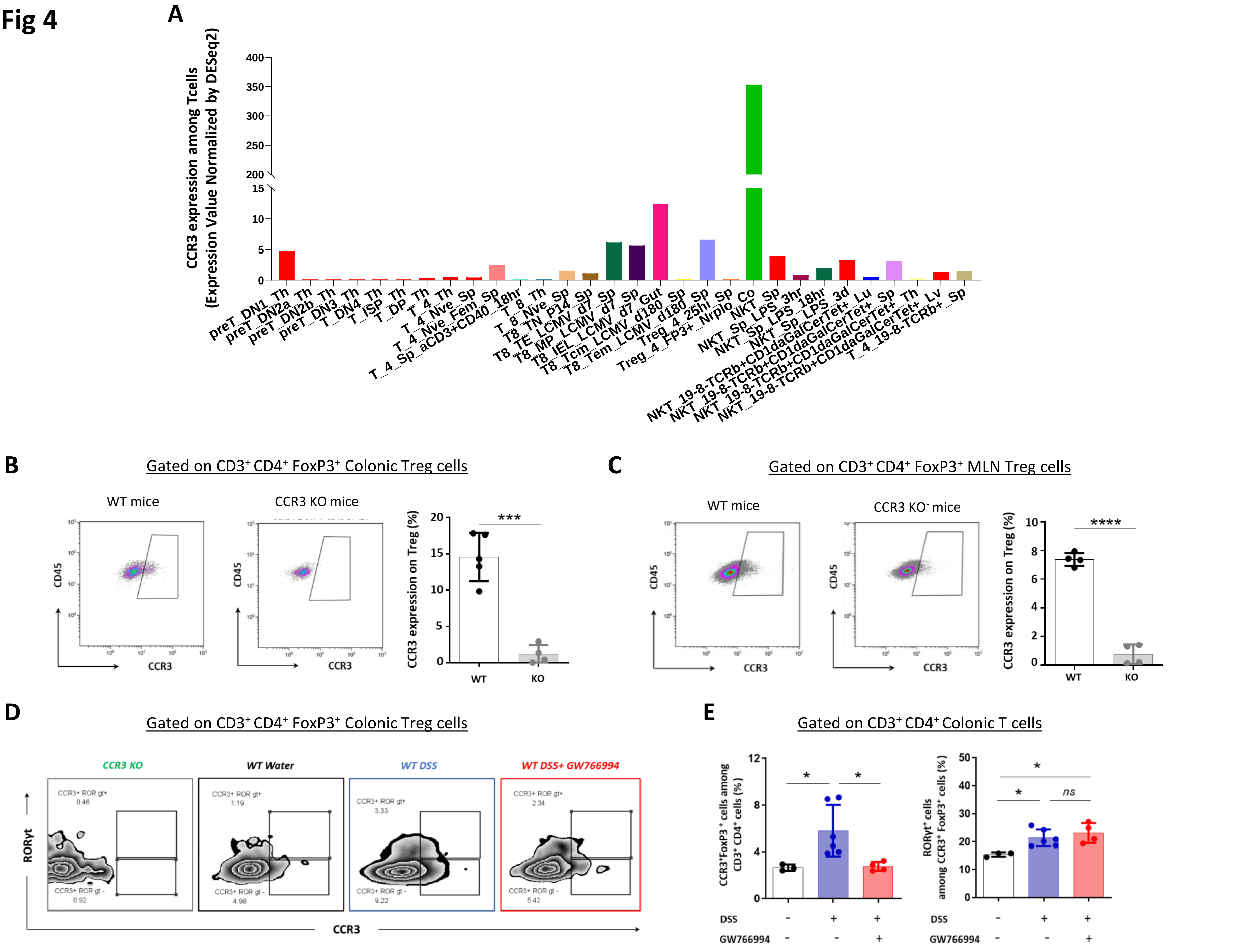
A
CCR3 expression among Tcells (Expression Value Normalized by DESeq2)
preT_DN1_Th
preT_DN2a_Th
preT_DN2b_Th
preT_DN3_Th
T_DN4_Th
T_ISP_Th
T_DP_Th
T_4_Th
T_4_Nve_Sp
T_4_Nve_Fem_Sp
T_4_Sp_aCD3+CD40_18hr
T_8_Th
T_8_Nve_Sp
T8_TN_P14_Sp
T8_TE_LCMV_d7_Sp
T8_MP_LCMV_d7_Sp
T8_IEL_LCMV_d7_Gut
T8_Tcm_LCMV_d180_Sp
T8_Tem_LCMV_d180_Sp
Treg_4_25hi_Sp
Treg_4_FP3+_Nrplo_Co
NKT_Sp
NKT_Sp_LPS_3hr
NKT_Sp_LPS_18hr
NKT_Sp_LPS_3d
NKT_19-8-TCRb+CD1daGalCerTet+_Lu
NKT_19-8-TCRb+CD1daGalCerTet+_Sp
NKT_19-8-TCRb+CD1daGalCerTet+_Th
NKT_19-8-TCRb+CD1daGalCerTet+_Lv
T_4_19-8-TCRb+_Sp
B
Gated on CD3+ CD4+ FoxP3+ Colonic Treg cells
WT mice
CCR3 KO mice
CD45
CCR3
CCR3 expression on Treg (%)
***
WT
KO
C
Gated on CD3+ CD4+ FoxP3+ MLN Treg cells
WT mice
CCR3 KO- mice
CD45
CCR3
CCR3 expression on Treg (%)
****
WT
KO
D
Gated on CD3+ CD4+ FoxP3+ Colonic Treg cells
CCR3 KO
WT Water
WT DSS
WT DSS+ GW766994
RORγt
CCR3
CCR3+ ROR gt+
0.46
CCR3+ ROR gt -
0.92
CCR3+ ROR gt+
1.19
CCR3+ ROR gt -
4.98
CCR3+ ROR gt+
3.33
CCR3+ ROR gt -
9.22
CCR3+ ROR gt+
2.34
CCR3+ ROR gt -
5.42
E
Gated on CD3+ CD4+ Colonic T cells
CCR3+FoxP3+ cells among CD3+ CD4+ cells (%)
RORγt+ cells among CCR3+ FoxP3+ cells (%)
*
ns
DSS - + +
GW766994 - - +

Fig Supp 1

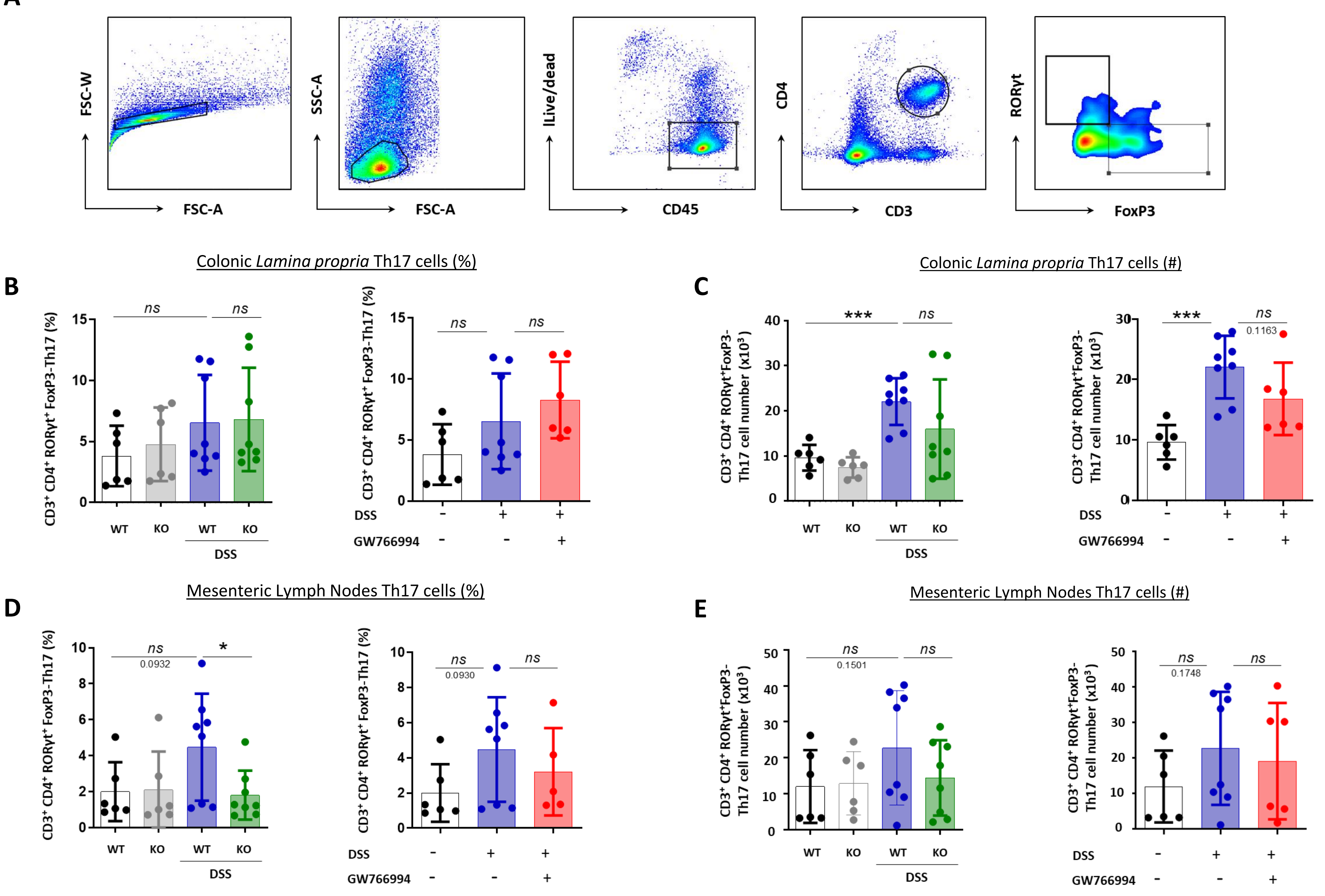

A
FSC-W
FSC-A
SSC-A
FSC-A
lLive/dead
CD45
CD4
CD3
RORγt
FoxP3
Colonic Lamina propria Th17 cells (%)
B
CD3⁺ CD4⁺ RORγt⁺ FoxP3-Th17 (%)
ns
ns
WT
KO
WT
KO
DSS
DSS
GW766994
Colonic Lamina propria Th17 cells (#)
C
CD3⁺ CD4⁺ RORγt⁺FoxP3- Th17 cell number (x10³)
***
ns
0.1163
Mesenteric Lymph Nodes Th17 cells (%)
D
*
0.0932
0.0930
Mesenteric Lymph Nodes Th17 cells (#)
E
0.1501
0.1748

Fig Supp 2

A

Colonic *Lamina propria* Treg/Th17 Ratio

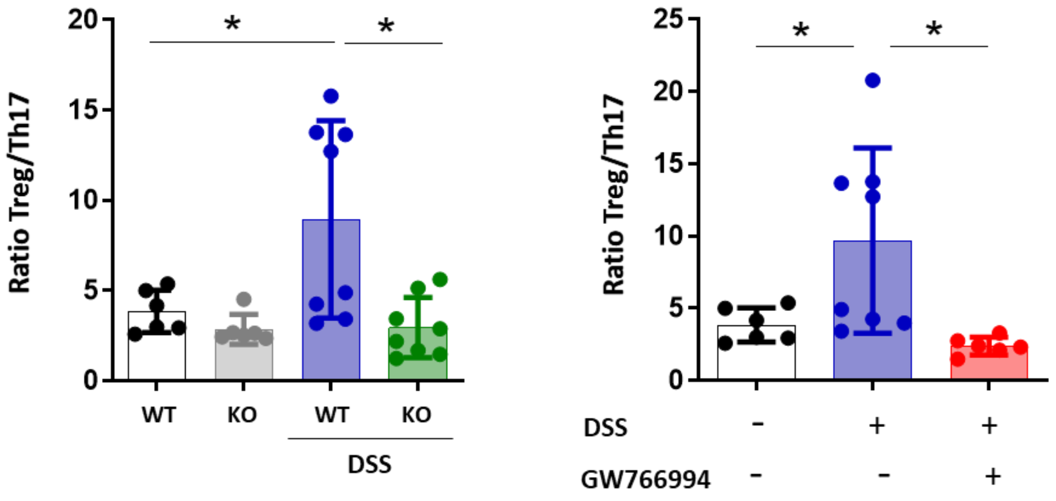

B

Mesenteric Lymph Nodes Treg/Th17 Ratio

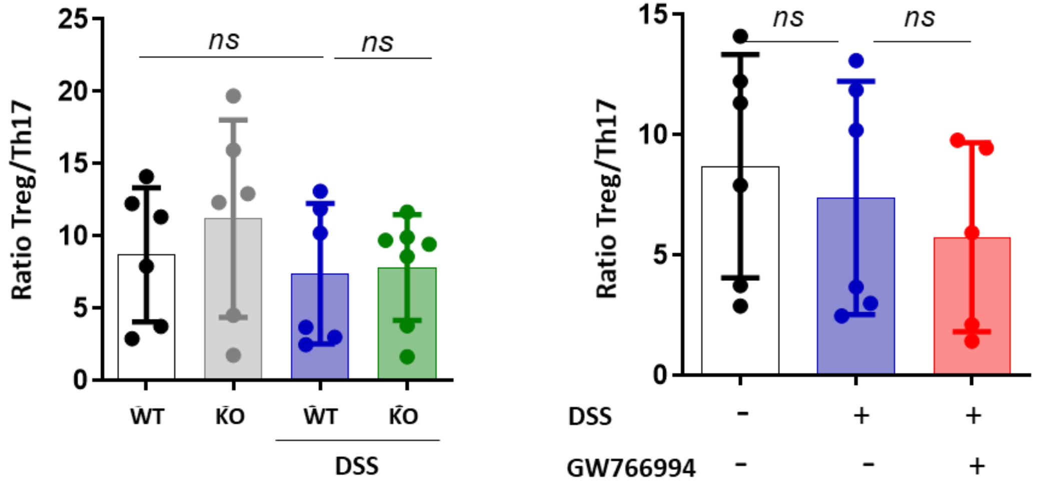

Supplement: Supplementary file 2 — Supplemental Figure 1. CRR3 inhibition does not exacerbate Th17 cell population after DSS challenge. (A) Flow cytometry dot plots showing Th17 gating strategy. (B) Frequency of CD3+ CD4+ RORγt+ Th17 cells isolated from colonic lamina propria of mice challenged or not with DSS. (C) Th17 cell count in colonic lamina propria of mice challenged or not with DSS. (D) Frequency of CD3+ CD4+ RORγt+ Th17 cells isolated from mesenteric lymph nodes (mLN) of mice challenged or not with DSS. (E) Th17 cell count in mLN of mice challenged or not with DSS. (n = 3/group). Data are expressed as mean ± SEM, ANOVA with Tukey posthoc test for multiple comparisonswas used for statistical differences between groups. n.s: non‐significant; * p < 0.05; ** p < 0.01 and *** p < 0.001. Supplemental Figure 2. CCR3 inhibition impairs Treg/Th17 balance in DSS‐induced colitis. (A) Treg/Th17 ratio in colon lamina propria of mice challenged or not with DSS. (B) Treg/Th17 ratio in mesenteric lymph nodes (mLN) of mice challenged or not with DSS. (n = 3/group). Data are expressed as mean ± SEM, ANOVA with Tukey posthoc test for multiple comparisonswas used for statistical differences between groups. n.s: non‐significant; * p < 0.05; ** p < 0.01 and *** p < 0.001. [file CTM2-11-e455-s001.pdf]
